# Peri-implant peripheral giant cell lesions: report of 13 new cases and comparative histological and immunohistochemical analysis with peripheral and central giant cell lesions

**DOI:** 10.4317/medoral.23088

**Published:** 2019-10-27

**Authors:** Thayná Melo de Lima Morais, Ciro Dantas Soares, José Manuel Aguirre Urizar, Javier Alberdi-Navarro, Oslei Paes de Almeida, Fábio Ramôa Pires

**Affiliations:** 1 MSc, Oral Pathology, Department of Oral Diagnosis, Piracicaba Dental School, University of Campinas, Brazil; 2 PhD, Oral Pathology and Medicine, Department of Stomatology II, University of the Basque Country (UPV/EHU), Leioa (Bizkaia), Spain; 3 PhD, Oral Pathology, Department of Oral Diagnosis, Piracicaba Dental School, University of Campinas, Brazil; 4 PhD, Oral Pathology, School of Dentistry, State University of Rio de Janeiro, Brazil

## Abstract

**Background:**

Few cases or peri-implant peripheral giant cell lesions (PGCL) have been reported in the literature. The aim of this study was to report 13 new cases of peri-implant PGCL and compare the expression of smooth muscle actin, Bcl-2 protein, GLUT-1, CD68, osteoprotegerin, receptor activator of nuclear factor kappa-B, Ki-67 and CD34 in these cases with PGCL and central giant cell lesions (CGCL).

**Material and Methods:**

Clinical data were retrieved from the laboratory records and histological analysis was performed using HE-stained slides. Immunohistochemical reactions for the above mentioned antibodies were performed and digitally scored.

**Results:**

Peri-implant PGCL mostly affected the posterior mandible of adult females. CD68 and Bcl-2 expressions were higher in conventional PGCL and CGCL than in peri-implant PGCL (*p*=0.033 for CD68 and *p*<.0001 for Bcl-2). Microvessel density was higher in conventional peripheral than in central and peri-implant PGCL (*p*=0.002). Proliferative index of the mononuclear cells showed no statistically significant differences comparing the three groups but it was higher in peri-implant PGCL.

**Conclusions:**

The current study demonstrated that peri-implant PGCL is more common in the posterior mandible of adult females. There were some differences in microvessel density, proliferative activity and expression of CD68 and Bcl-2 among conventional PGCL, peri-implant and CGCL. Further studies are encouraged to better understand these early findings.

** Key words:**Giant cell lesion, giant cell granuloma, peripheral, dental implants, immunohistochemistry.

## Introduction

Peripheral giant cell lesions (PGCL) are relatively common proliferative growths that affect mainly the lower posterior gingiva and edentulous areas of the alveolar mucosa (1). It is well accepted that these lesions are reactive processes microscopically characterized by a proliferation of multinucleated giant cells (possibly osteoclast-like type derived from the periodontal ligament) associated with inflammatory mononuclear cells, mesenchymal cells, and variable amounts of blood vessels, hemorrhage and fibroblasts (1,2). Local irritative factors and chronic trauma are the main etiological factors recognized to date (2). Central giant cell lesions (CGCL) are intraosseous conditions that occur mainly in the mandible and are histologically similar to PGCL (3). The exact pathogenesis of CGCL is still controversial and its etiology has not been fully elucidated, but some lesions histologically identical to CGCL are associated with other entities, such as hyperparathyroidism, cherubism, Noonan syndrome, and type-1 neurofibromatosis (4,5).

Almost all PGCL are associated with teeth or arise in edentulous areas of the alveolar mucosa, but there are also some few reported cases of peri-implant PGCL (1). Conventional PGCL, peri-implant PGCL and CGCL are histologically similar, but their pathogenesis remains poorly understood. The immunoprofile of PGCL and CGCL has been widely investigated and expression of numerous markers for proliferative activity, osteoclast metabolism, angiogenesis and apoptosis have been studied in these lesions (6-9). However, there is no previously published comparative study including peri-implant PGCL and it is not known whether the expression of metabolism markers and proteins involved in osteoclast activation pathways and apoptosis can be different when comparing these 3 groups. Therefore, the aim of the present study was to describe the clinicopathological features of a series of peri-implant PGCL and to compare the histological and immunohistochemical profile of conventional and peri-implant PGCL and CGCL.

## Material and Methods

The sample consisted of 36 specimens retrieved from the files of the Oral Pathology Laboratory, School of Dentistry, State University of Rio de Janeiro, Brazil and from the Oral & Maxillofacial Pathology Laboratory, University of the Basque Country/EHU, Leioa, Spain. After analysis of the integrity of the specimen and its representativeness, the cases were selected and grouped in CGCL (n=10), conventional PGCL (n=13) and peri-implant PGCL (n=13). Clinical, radiological and gross specimen data from each case were obtained from the laboratory registries and histological features were described after analysis of five-µm HE-stained sections. This study was approved by the Institutional Board Review of the *Pi*racicaba Dental School, University of Campinas (CEP/FOP; process number 70503317.8.0000.5418).

Immunohistochemical (IHC) reactions were performed using 3-µm sections deparaffinized in xylene and hydrated in graded series of alcohol. Antigen retrieval was performed with a citric acid solution (pH 6.0) (α-Smooth muscle actin - α-SMA, Bcl-2, Glucose transporter 1 - Glut-1, CD68, and CD34) or EDTA/Tris solution (osteoprotegerin – OPG, receptor activator of nuclear factor kappa-B – RANK, and Ki-67) and pressure cooker. The activity of the endogenous peroxidase was blocked with one 15-minute bath of 10% hydrogen peroxide. The sections were incubated for 2 hours with the primary antibodies against α-SMA (clone 1A4, dilution 1:400, positive control – endometrium, Dako, Carpinteria, CA, USA), Bcl-2 (clone 124, dilution 1:50, positive control – lymph node, Dako, Carpinteria, CA, USA), Glut-1 (polyclonal, dilution 1:100, positive control – fibrous hyperplasia, BioSystems, Evry cedex, France), CD68 (clone PG-M1, dilution 1:400, positive control – mucocele, Dako, Carpinteria, CA, USA), OPG (clone ab183910, dilution 1:100, positive control – newly formed bone tissue, Abcam, Cambridge, MA, USA), RANK (clone 64c1385, dilution 1:100, positive control – newly formed bone tissue, Abcam, Cambridge, MA, USA), CD34 (clone QBEnd-10, dilution 1:50, positive control – pyogenic granuloma, Dako, Carpinteria, CA, USA) and Ki-67 (clone MIB-1, dilution 1:100, positive control – lymphoma, Dako, Carpinteria, CA, USA). After that, the sections were incubated with the super-sensitive non-biotin based IHC visualization system (AdvanceTM HRP Kit – DakoCytomation, Carpinteria, CA, USA). Diaminobenzidine tetrahydrochloride (DAB, Sigma, St. Louis, MO, USA) was used as chromogen and was followed by counterstaining with Carazzi’s hematoxylin.

All HE-stained and IHC slides were scanned into high-resolution images using the Aperio Scanscope CS Slide Scanner (Aperio Technologies Inc., Vista, California, USA). For α-SMA, Bcl-2, Glut-1, CD68, OPG and RANK, the Positive *Pi*xel Count v9 (Aperio Technologies Inc.) algorithm classified staining in negative, weak-positive, medium-positive and strong-positive; five distinct medium-power fields were evaluated for each case. The final score of each marker for each lesion was calculated as the sum of the percentage of each category multiplied by their intensity scores using the following formula: [tumor score = (percentage weak × 1) + (percentage moderate × 2) + (percentage strong × 3)]. The results ranged from 100 to 300, which was in agreement with previous study from our group (10). For Ki-67, the Nuclear Algorithm (Aperio Technologies Inc.) detected the total, negative and positive nuclear staining, generating an index of the positivity (in %), and it was also calculated for the surface epithelium. Microvessel density, assessed by counting the total of CD34-positive vessels, was performed by the software Microvessel Analysis Algorithm (Aperio Technologies Inc.). The ratio of giant/mononuclear cells was also calculated for each case. All histomophometric and digital-IHC analyzes were carried out in five high-power fields (×40 magnification) and the mean was calculated.

Statistical Package for Social Sciences (SPSS) software version 22 (IBM, Chicago, IL, USA) was used for statistical analysis. Normality was analyzed with the Kolmogorov-Smirnov test. Kruskal-Wallis test was applied to compare the means from all scores, cellular proliferative index and microvessel density. Data were also examined through the Pearson correlation test. The level of significance was considered 5% (*p* ≤ 0.05) for all analyses.

## Results

Peri-implant PGCL affected mostly females (11 cases – 85%) and mean age of the patients was 57.5 years (ranging from 29 to 73 years old). Ten cases affected the mandible (77%) and 70% of the cases were located in the posterior region. Clinical aspect of the lesions was described as a reddish/purplish growth in 62% of the cases and most cases presented less than 15 mm in size in its greater diameter. Clinical diagnosis included mostly pyogenic granuloma (in 10 cases) and PGCL was suspected in only two cases. Clinical and demographical characteristics comparing the three groups are shown in Table 1.

**Table 1:**
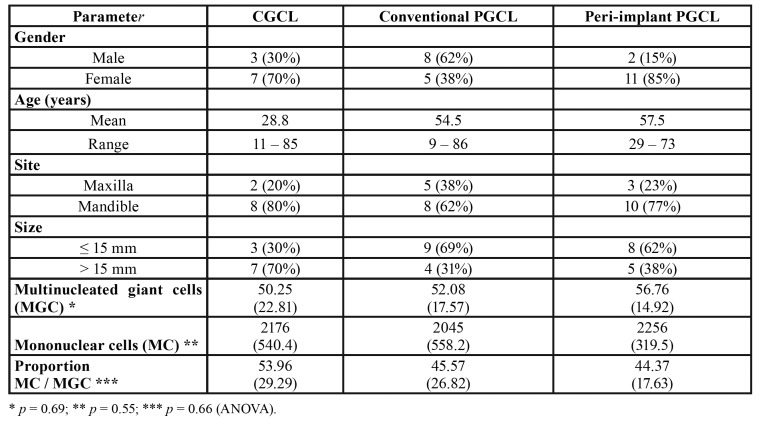
Clinical and demographic data and mean number (standard deviation) of multinucleated giant cells (MGC) and mononuclear cells (MC) per high-power field, and proportion of mononuclear/giant cells in central giant cell lesions (CGCL) and conventional and peri-implant peripheral giant cell lesions (PGCL).

CGCL and peri-implant PGCL demonstrated a predilection for females while conventional PGCL were more common in males. Mean age of the patients affected by PGCL was higher than the mean age of the CGCL-affected patients. All three groups were more common in the mandible and most PGCL were smaller than the CGCL (Table 1).

**Figure 1:**
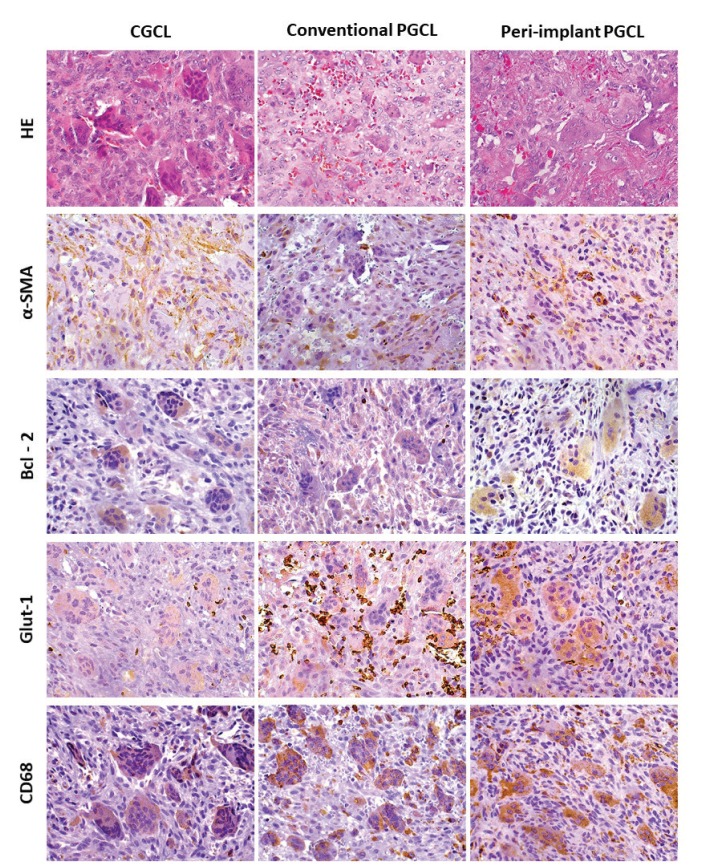
Histological features and immunohistochemical expression of α-SMA, Bcl-2, Glut-1 and CD68 in CGCL (central giant cell lesions), conventional and peri-implant PGCL (peripheral giant cell lesions) (All 400x magnification).

All lesions showed a similar histological pattern. Conventional and peri-implant PGCL were mostly covered by a keratinized stratified squamous epithelium, with a higher frequency of ulceration in peri-implant PGCL. The lamina propria was composed by a loose connective tissue and the deeper parts of the stroma showed a proliferation of multinucleated giant cells interspersed by a component of ovoid and spindled shaped mesenchymal cells and numerous small blood vessels. In CGCL, a spindle cell proliferation was also present and multinucleated giant cells were observed in direct contact with bone trabeculae. Multiple areas of hemorrhage and presence of hemosiderin were also common in CGCL (Fig. 1). Mean number of multinucleated giant cells per high-power field (×40 magnification) was 50.25, 52.08 and 56.76 for CGCL, conventional PGCL and peri-implant PGCL, respectively. Proportion of ovoid/spindled mononuclear mesenchymal cells to multinucleated giant cells was 53.96, 45.57 and 44.37 for CGCL, conventional PGCL and peri-implant PGCL, respectively. Differences were not statistically significant (Table 1).

The IHC features showed that α-SMA was expressed in blood vessels and myofibroblasts in all groups. Bcl-2 demonstrated a variable expression in mononuclear cells and multinucleated giant cells. Several cases showed a weak expression of Bcl-2 in multinucleated giant cells. Ulcerated conventional and peri-implant PGCL showed a higher number of inflammatory cells positive for Bcl-2 close to areas of ulceration. Glut-1 was expressed in the cytoplasm of few multinucleated giant cells in all groups and surface epithelium in conventional and peri-implant PGCL. CD68 was expressed mainly in the cytoplasm of the multinucleated giant cells and some disperse mononuclear cells. RANK and OPG demonstrated a cytoplasmic expression in fusiform and mononuclear cells, and some giant cells were also strongly positive for both markers. CGCL presented higher expression of RANK than conventional and peri-implant PGCL, while OPG was more expressed in peri-implant PGCL than in conventional PGCL and CGCL. CD34 was expressed mainly in the endothelial cells of large and small vessels and occasionally in dispersed fusiform cells, compatible with fibroblasts/myofibroblasts. Fig. 1 and Fig. 2 show some histological features of the studied lesions as well as the comparative immunoexpression of α-SMA, Bcl-2, Glut-1, CD68, RANK, OPG and CD34.

The proliferative index of the mononuclear ovoid/spindled mesenchymal cells, assessed by Ki-67-positive nuclei was 5.33%, 5.24% and 6.49% for CGCL, conventional PGCL and peri-implant PGCL, respectively (Fig. 2).

**Figure 2:**
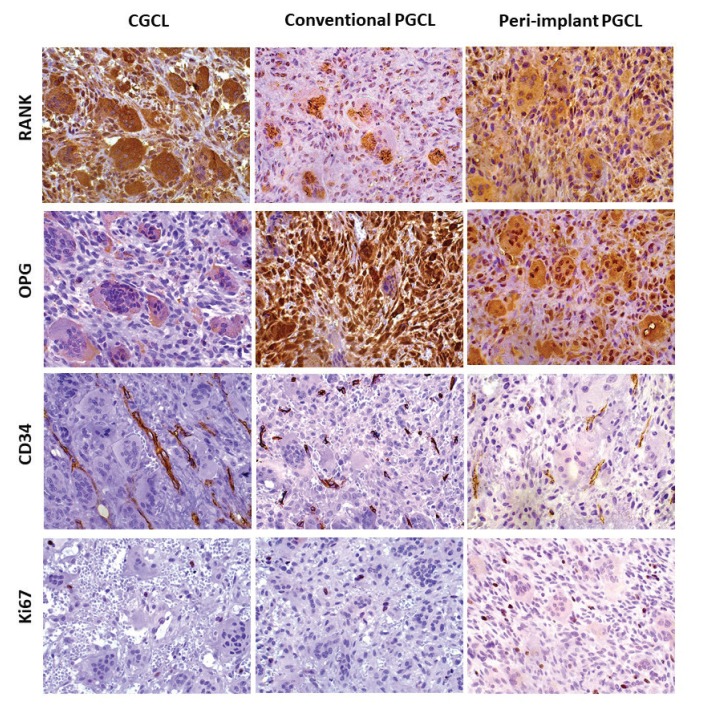
Immunohistochemical expression of RANK, OPG, CD34 and Ki67 in CGCL (central giant cell lesions), and conventional and peri-implant PGCL (peripheral giant cell lesions) (All 400x magnification).

Overall, in peripheral lesions the proliferative index of the surface epithelium was 11.16% for conventional PGCL and 22.49% for peri-implant PGCL. The epithelial proliferative index in non-ulcerated conventional PGCL was 11.35% (10/13 cases) and ulcerated was 10.53% (3/13 cases). Regarding the peri-implant PGCL the values were 18.31% for non-ulcerated lesions (3/13 cases) and 23.75% for ulcerated lesions (10/13 cases).

Mean scores from the digital analysis for all studied markers are shown in Fig. 3.

**Figure 2:**
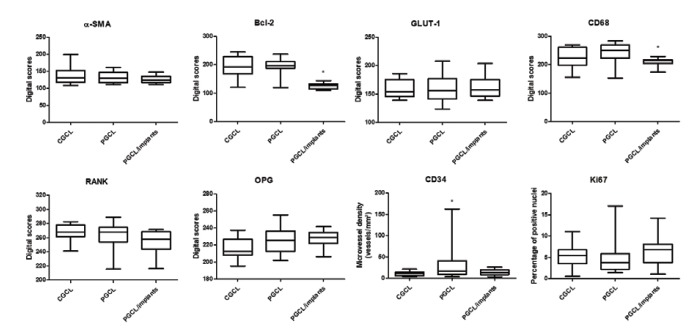
Comparison of the expression of the studied markers in the three groups: CGCL (central giant cell lesions), PGCL (conventional peripheral giant cell lesions) and PGCL/implants (peri-implant pe ripheral giant cell lesions) (Statistically significant differences are shown by asterisks).

CD68 and Bcl-2 scores were higher in conventional PGCL and CGCL than in peri-implant PGCL (*P*=0.033 for CD68 and *P*<0.0001 for Bcl-2). Microvessel density was higher in conventional PGCL than in CGCL and peri-implant PGCL (*P*=0.002). The α-SMA, Glut-1, RANK and OPG scores demonstrated a similar expression in the three groups, without any statistically significant differences. Correlation tests demonstrated a positive correlation between microvessel density and the expression of CD68 (r= 0.360, *P*=0.034) and OPG (r=0.382, *P*=0.024).

## Discussion

Most studies have demonstrated that oral mucosal and gnathic giant cell lesions show a similar histological pattern and are considered reactive entities, although they sometimes present a local aggressive clinical course (3-5). They can affect the gnathic bones and the gingiva/alveolar mucosa and can rarely be associated with dental implants (1,3,11). Several studies have compared the clinicopathological and immunohistochemical features of PGCL and CGCL, mostly directed to proliferative pattern and histogenetic origin of the multinucleated giant cells and mononuclear spindle cells (6-9). As far as we know, this is the first study focusing these histological and immunohistochemical comparisons including peri-implant PGCL.

There are less than 30 cases of peri-implant PGCL reported in the English-language literature since its first description by Hirshberg *et al*. in 2003 (11-25). Apart from isolated case reports, only one previous comparative study has included 9 cases of peri-implant PGCL (25). The present series is the largest reported up to now and possibly includes a representative comparative profile of this condition. The previously published cases reinforce the present results showing that peri-implant PGCL is more common in the posterior mandible of females in the fifth to sixth decades of life (11-25). This distribution could be explained by the fact that dental implants are more commonly installed in the mandible of older females. In the present sample, CGCL and peri-implant PGCL occurred more commonly in the mandible of females, in accordance with previous studies (18,22,23). Both conventional and peri-implant PGCL affected older patients in comparison with CGCL, also in accordance with the literature (4,5,18). It is interesting to notice that most cases were clinically interpreted as pyogenic granulomas, but clinicians should be aware that PGCL can be also associated with dental implants.

The present results showed that both the mean number of multinucleated giant cells per high-power field and the proportion of ovoid/spindled mononuclear mesenchymal cells to multinucleated giant cells were similar for CGCL, conventional PGCL and peri-implant PGCL. One previous study showed, however, a significantly lower number of giant cells and a lower density of mesenchymal cells in peri-implant PGCL in comparison with conventional PGCL (25). It is possible that the low number of cases could bias these findings and more studies including larger samples are necessary to understand these initial observations.

CD68 is a transmembrane glycoprotein widely used as a specific marker of cells of the monocyte-macrophage lineage, such as monocytes, histiocytes and osteoclasts. We observed a higher CD68 expression score in CGCL and conventional PGCL than in peri-implant PGCL. Conventional PGCL demonstrated a higher CD68 expression score in comparison with CGCL, although not statistically significant, in accordance with previous results (8). An intriguing result from our study was the higher number of giant cells but lower expression of CD68 in peri-implant PGCL in comparison with the other two groups. It has been demonstrated that immature mononuclear cells derived from the bone marrow do not express CD68, and their stimulation by macrophage colony-stimulating factor is time-dependent (26), so osteoclasts in these lesions can be derived exclusively from the bone in the area adjacent to the implant. CD68-deficient osteoclasts have demonstrated lower resorption activity and, in some instances, stimulates osteoblasts (26). Thus, we can suggest that CD68-deficient osteoclasts are more common in peri-implant PGCL due to the absence of periodontal ligament. However, we were not able to explain if osteoclasts of conventional PGCL have higher resorption activity and, consequently, demonstrate higher CD68-expression.

Macrophages are potent angiogenic stimulators by producing several angiogenic factors and our results showed a positive correlation between the expression of CD68 and microvessel density. Conventional PGCL showed both a higher microvessel density and a higher score of CD68-positive cells than peri-implant PGCL; one explanation for these findings could be that conventional PGCL is exposed to continuous local irritative factors from the periodontal ligament or local trauma, showing an increase in the number of blood vessels and, consequently, a higher microvessel density. Higher microvessel density has been also associated with a more aggressive phenotype of CGCL (27), but we were not able to evaluate this possible association due to the methods used in the present study.

Several components of the B-cell lymphoma family, particularly Bcl-2, have been involved in the apoptotic pathway, acting as positive or negative regulators of this process (28). It is well established that Bcl-2 facilitates cell survival, playing an important role in tumor initiation and maintenance (9,28). Expression of Bcl-2 was previously demonstrated in multinucleated giant cells from both PGCL and CGCL (9,28) and, in the present study, its expression was significantly lower in peri-implant PGCL in comparison with the other groups. It seems that the influence of apoptotic factors is less important in the growth and maintenance of peri-implant PGCL than conventional PGCL, but further investigations with larger samples and including other proteins involved in the apoptotic pathway are encouraged to clarify the importance of these findings.

RANK pathway is directly involved in the dynamics of osteoclastogenesis, playing an important role in osteoclast proliferation, activation and apoptosis (29). On the other hand, OPG acts as an inhibitor of the osteoclast activity. In the current study, RANK and OPG presented similar digital scores in all three groups, suggesting that these markers exert an important role in the histogenesis of multinucleated giant cells in giant cell lesions independent from its origin (peripheral or central) and relationship to periodontal ligament or not.

Expression of α-SMA was similar in the three groups suggesting that the presence of cells with myofibroblastic phenotype is constant in GCL and is not dependent on origin and relationship to periodontal ligament. Glut-1 is a molecule involved in the basal glucose uptake and, consequently, plays an important role in the cellular metabolism, energy production and intracellular glucose concentration levels (30). Its expression was also similar in the three groups and apparently does play a specific origin-dependent role in the pathogenesis of giant cell lesions.

Comparison of the proliferative index of the mononuclear cells from the three groups showed no statistically significant differences. However, the proliferative index was higher in peri-implant PGCL than in conventional PGCL and CGCL. The higher proliferative index of the peri-implant PGCL could be associated with the higher frequency of ulceration of the surface epithelium seen in this group and seems to be not associated with apoptotic pathways. Peri-implant PGCL have demonstrated higher intralesional and epithelial proliferative index in comparison with conventional PGCL and CGCL, together with lower expression of Bcl-2 compared with the other lesions.

In conclusion, the results of the current study showed that peri-implant PGCL are more common in the posterior mandible of adult females. We have also demonstrated some differences in microvessel density, proliferative activity and expression of CD68 and Bcl-2 among conventional PGCL, peri-implant PGCL and CGCL. Although this series is the largest reported up to now, the present study has some limitations in data interpretation such as the limited number of cases and the difficulties in obtaining information on follow-up, limiting the discussion about biological behavior of peri-implant PGCL. Further studies with larger series with follow-up and inclusion of additional histogenetic markers are encouraged to better understand these early findings.
